# Decrease in incidence of proximal femur fractures in the elderly population during the Covid-19 pandemic: a case–control study

**DOI:** 10.1186/s12891-022-05016-2

**Published:** 2022-01-17

**Authors:** Maria Oulianski, Philip J. Rosinsky, Ariel Fuhrmann, Ruslan Sokolov, Roberto Arriola, Omri Lubovsky

**Affiliations:** Department of Orthopedic Surgery, Barzilai Medical Center, Ashkelon, Israel

**Keywords:** Covid-19, Coronavirus, Hip fractures, Trauma, Elderly patient, Proximal femur fracture

## Abstract

**Background:**

The World Health Organization classified Covid-19 as a pandemic during the first months of 2020 as lockdown measures were implemented globally to mitigate the increasing incidence of Covid-19-related morbidity and mortality. The purpose of this study was to evaluate the effect of national lockdown measures on proximal femur fracture epidemiology. Our hypothesis was that due to the prolonged period of stay-at-home orders, we would observe a decrease in the incidence of proximal femur fractures during the years 2020–21.

**Methods:**

A retrospective case–control study of 2784 hip fractures admitted to the emergency department at one hospital between January 1, 2010, and March 31, 2021, was conducted. Cases were stratified weekly, and an analysis was conducted comparing cases occurring during government-imposed lockdown periods of 2020–21 to corresponding periods during 2010–2019. Furthermore, the trend of cases throughout the year of 2020 was observed.

**Results:**

Of all proximal femur fracture cases included, 2522 occurred between 2010–2019 and 261 during the Covid-19 period. There was no significant difference in age (81.95 vs. 82.09; *P* = 0.78) or gender (*P* = 0.12). There was a total decrease of 21.64% in proximal femur fracture per week during the entirety of the Covid-19 pandemic period compared to the previous years (3.64 ± 1.99 vs. 4.76 ± 0.83; *P* = 0.001). During all three lockdown periods, there was a significant decrease in proximal femur fracture cases per week (3.55 ± 2.60 vs. 4.87 ± 0.95; *P* = 0.04), and the most pronounced decrease occurred during the third lockdown period (2.89 ± 1.96 vs. 5.23 ± 1.18; *P* = 0.01).

**Conclusion:**

We observed a total decrease in the number of proximal femur fractures occurring during the Covid-19 era compared to previous years and specifically a decrease of cases occurring during the government-imposed lockdown periods. The decrease in cases was more pronounced during the second and third lockdown periods.

## Introduction

The Covid-19 pandemic was caused by the Coronavirus-2 of Severe Acute Respiratory Syndrome (SARS-CoV-2). First reported in December 2019 in Wuhan, China, the virus rapidly spread worldwide until the World Health Organization (WHO) declared a pandemic on January 30, 2020 [1, 2]. In the United States, the first cases of Covid-19 were described at the beginning of January 2020, and by the end of January 2020, first cases were being reported in Europe [3, 4]. The rapid spread of Covid-19 called for immediate action from policy-makers, and in many countries, including Israel, lockdown measures were implemented.

In Israel, the first case of Covid-19 was confirmed on February 21, 2020. Subsequently, the Israeli Ministry of Health (MOH) put into effect steps to mitigate the spread of the virus. By the beginning of March, all Israelis returning from abroad were instructed to begin a 14-day home-isolation period upon arrival, and by mid-March, further gradual lockdown measures were imposed, including restrictions on movement, reduction of working capacity, and closure of the educational system for all ages [5–7]. By March 25, a full lockdown was imposed. The initial lockdown measures were successful in drastically reducing Covid-19 cases. However, throughout 2020 the number of cases fluctuated, with peaks occurring in August and November 2020, resulting in a second (09/2020) and third (12/2020) lockdown. On December 25, 2020 Israel initiated a national program for vaccination of the elderly population as well as healthcare workers, which ultimately succeeded in drastically lowering cases of Covid-19 [8].

The elderly population has been recognized as the group with the highest risk for morbidity and mortality due to Covid-19. Elderly patients suffering from comorbidities have been shown to be at an especially high risk for severe acute respiratory distress syndrome (ARDS), with mortality rates reaching up to 45% [9–12]. Similarly, this same population is the most at risk for suffering from proximal femur fractures (PFFs), including femoral neck fractures and pertrochanteric fractures [13, 14].

Osteoporotic fragility fractures, and specifically PFFs, occur in increasing numbers due to ageing of the population, with over 500,000 annual cases in Europe [15]. Worldwide, PFFs are estimated to reach 2.6 million cases per year by 2025 [16, 17]. These patients represent a significant health care burden with high rates of post-injury morbidity and mortality. These are related to complications associated with the lack of mobility in this population and concomitant medical conditions. At a period of increased health system burden due to the concurrent Covid-19 pandemic, the burden of PFFs on the health-system is even more critical [16, 18–21].

During the Covid-19 related lockdowns, the limited ability of patients to leave their private homes reduced outdoor activity, thus potentially decreasing the risk of falling. On the other hand, some previous studies have demonstrated that most elderly patients fall in their households from a standing height [22, 23]. Therefore, the purpose of this study was to evaluate whether government-imposed lockdown in Israel would impact the occurrence of proximal femur fractures during the years of 2020–21. We hypothesized that we would observe a significant effect of the lockdown periods on the incidence of PFFs.

## Methods

Findings were reported according to the STrengthening the Reporting of OBservational studies in Epidemiology (STROBE) checklist for retrospective cohort studies [24]. Data were retrospectively extracted from the medical records of all patients treated for hip fractures presenting to the emergency department (ED) of a tertiary medical center in the south region of Israel between 2010 and March 31, 2021. The range of dates was chosen to include the years of 2020–21, during which the Covid-19 pandemic occurred, as well as the 10 years prior, in order to serve as a comparison.

Patients were selected for inclusion based on a presentation to the ED with a proximal femur fracture identified using the International Classification of Diseases, 9th edition (ICD-9) diagnosis of hip fracture (820.xx). The electronic patient files were searched, and the following demographic data were obtained: age; gender; country of birth; whether residing at home, skilled nursing facility, or other; date of hospitalization, and country of birth. Further, the type of fracture was classified based on radiographic images upon presentation, as transcervical, base of neck, or pertrochanteric fractures. Patients were included if they presented with the aforementioned fractures and were aged over 65. Patients presenting with fractures following high-energy mechanisms, polytrauma, periprosthetic fractures, and fractures of the middle and distal third of the femur were excluded.

**Fig. 1:**
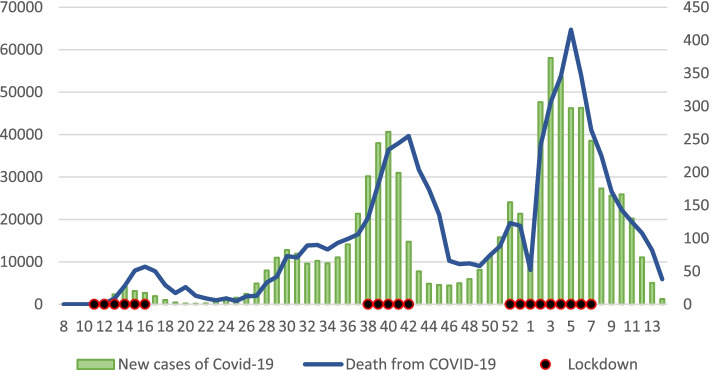
Covid-19 Cases and Weekly Mortality**.** The left y-axis and the green columns refer to new cases by weeks during 2020 and 2021. The right y-axis and blue line refer to weekly mortality rates. Black dots represent weeks during which complete lockdown measures were implemented

To demonstrate the fluctuations and estimate the severity of the Covid-19 pandemic throughout the course of the year 2020 in Israel, weekly new case counts and weekly mortality rates attributed to Covid-19 were collected. Weeks were counted based on the International Organization for Standardization (ISO) week calendar system, which divides the year into 53 weeks and the first 14 weeks of 2021. This data was available through the Covid-19 Cases Data from Humanitarian Data Exchange (HDX) platform [25], the WHO organization [26], and the MOH [27]. Lastly, data on lockdown measures implemented in Israel were collected from government announcement reported in the media outlets [28–30]. Data regarding Covid-19 new cases, mortality, and lockdown measures are presented in Fig. 1.

The incidence of hip fractures throughout the study period was compared based on the following data periods: the pre-Covid-19 era, defined between the years of 2010 and the initial weeks of 2020, and the Covid-19 era defined as the period from the first lockdown period and up to the 13^th^ week of 2021. During 2020–21 there were three separate lockdown periods; weeks 11–16 (A), weeks 38–42 (B), and weeks 52–60 (including the first 6 weeks of 2021) (C).

Categorical variables were reported as frequencies and percentages and compared using Pearson’s chi-square test. Continuous variables were reported as a median and interquartile range, or mean and standard deviation, and compared using the Student’s t-test or analysis of variance (ANOVA), as appropriate. Normality was assessed using the Kolmogorov–Smirnov test. A *p*-value < 0.05 was considered statistically significant. Data analysis was performed using IBM SPSS Statistics for Windows (Version 25.0, Armonk, NY). Diagrams of curves were drawn with the use of Microsoft Excel 365 (Version 2101, Microsoft Corp., Redmond, WA).

## Results

During the study period, 2784 patients presented with proximal femur fractures and met inclusion and exclusion criteria. The average patient age was 81.97 ± 7.62. Demographic data regarding the entire cohort, as well as a comparison of the pre-Covid-19 and Covid-19 cohorts are presented in Table 1.

**Table 1:** Demographic data

	**Total**	**2010–2019**	**2020–2021**	***p*-value**
Cases	2784	2522	261	
Age: Mean (SD)	79.68 (10.64)	81.95 (7.54)	82.09 (8.38)	0.78
Gender—Female	1966 (70.61%)	1792 (71.05%)	174 (66.67%)	0.12
Fracture type: intracapsular	1287 (46.23%)	1166 (46.23%)	121 (46.36%)	0.98

*SD* Standard deviation

**Fig. 2:**
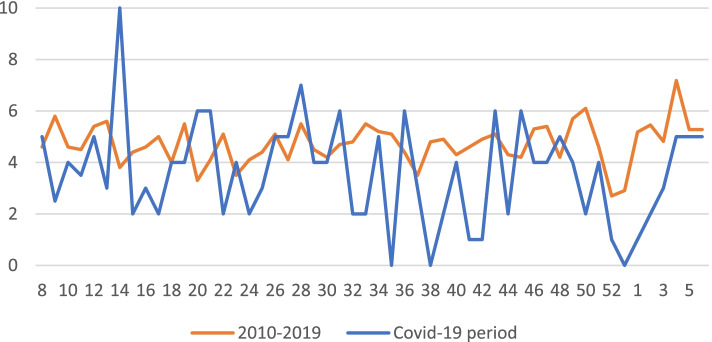
Weekly distribution of new cases of proximal femur fractures. Comparing the 2010–2019 and Covid-19 periods

There were no statistically significant differences in age, gender, and fracture type between pre-Covid-19 and Covid-19 cohorts. There was a total decrease of 21.64% in PFF per week during the entirety of the Covid-19 pandemic period compared to pre-Covid-19 period (3.64 ± 1.99 vs. 4.76 ± 0.83; *P* = 0.001), Fig. 2.

**Fig. 3:**
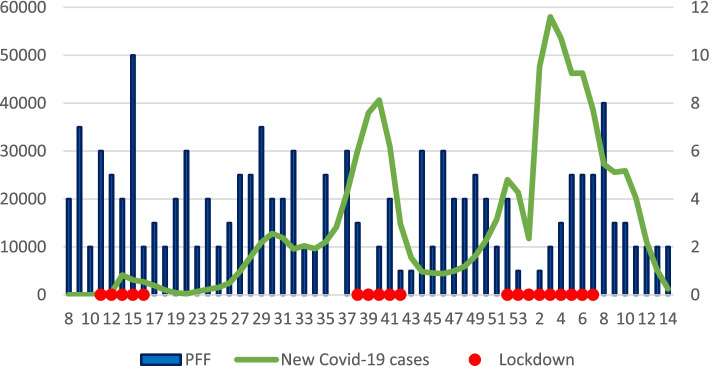
New Covid-19 Cases and Proximal Femur Fractures. The x-axis represents weeks, the right y-axis depicts proximal femur fractures, and the left y-axis depicts new cases of Covid-19

When comparing the period before appearance of Covid-19, from January 2020 to week 10 of 2020, to the corresponding weeks of 2010–2019, there was no significant difference in the number of PFF per week (5.20 ± 2.90 vs. 5.26 ± 1.29; *P* = 0.95). However, when comparing lockdown periods during 2020–21 to the corresponding weeks in 2010–19, statistically significant differences were found, with fewer cases occurring during lockdown priod. During lockdown A there were 5.83 ± 2.85 cases per week compared to 4.72 ± 0.67 cases in 2010–2019 (*P* = 0.37). During lockdown B, there were 2.00 ± 1.58 cases per week compared to 4.42 ± 0.56 cases in 2010–2019 (*P* = 0.02). During lockdown C, there were 2.89 ± 1.96 cases per week compared to 5.23 ± 1.18 cases in 2010–2019 (*P* = 0.01) (Fig. 3).

**Fig. 4:**
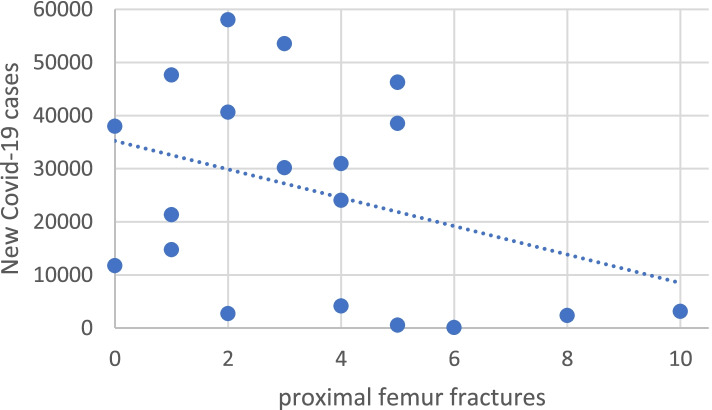
Correlation of new cases of Covid-19 and proximal femur fractures. Weekly number of proximal femur fractures during lockdown periods (*P* = 0.07)

A moderate correlation was found between new cases of Covid-19 and incidence of PFFs during lockdowns; thus, as the amount of new cases of Covid-19 increases, we can observe lower weekly PFFs (R = -0.35; *P* = 0.07) (Fig. 4).

Altogether, from week 12 (beginning of the first lockdown) till the beginning of March 2021, including all three lockdown periods, there was an average of 3.55 ± 2.60 cases per week compared to 4.87 ± 0.95 cases in the corresponding time period in 2010–2019 (*P* = 0.04).

## Discussion

The current study demonstrated the impact of the Covid-19 pandemic on the occurrence of PFFs. Specifically, the results show a decrease in the occurrence of these fractures during the lockdown periods, as well as an impact on the cases occurring during the entire year of 2020 and the beginning of 2021. Furthermore, during weeks without lockdown measures imposed, no such decrease was observed. This further implies that the reduction observed is not due to other confounding factors, such as a change in medical management or a change in the catchment area demographics.

Previous studies have demonstrated conflicting findings regarding the effect of Covid-19 on the incidence of PFFs [31–35]. Arafa et al. demonstrated a significant increase in the amount of PFFs during the pandemic period (61.7%). In contrast, Maniscalco et al. examined the period of February to March 2019 in comparison to the corresponding months of 2020 and demonstrated a decrease of 28.4% (169 vs. 121) in PFF incidence; similar results were demonstrated by Slullitel et al. [31, 32, 34] Compared to these studies, our present study encompassed the entire year of 2020 and included a larger patient cohort. Furthermore, our study included a sub-analysis based on the presence of lockdown measures. This analysis further accentuated the effect of Covid-19 on the incidence of PFFs.

Lim et al. conducted a systematic review and meta-analysis on occurrence of orthopedic fractures during the Covid-19 pandemic. They found that, during the pandemic, most of the fractures occurred at home due to domestic accidents with a decrease in sports-related injuries, as would be expected during periods of extended stay-at-home [36]. Dhillon et al. and Zhu et al. similarly showed that in the elderly population during the Covid-19 pandemic, home-related, low-energy falls were the most common fracture mechanism [37, 38].

We have shown a trend of decreased number of PFFs during the lockdown periods, suggesting that the new lifestyle and motion restriction may contribute to the decrease in fracture frequency. First, when the elderly population stays in a familiar environment, they maintain routine and are subjected to less unexpected and unfamiliar conditions. This in turn may reduce the likelihood of falls and therefore the incidence of PFFs. Second, as these fractures may also be due to a bone fatigue there is a possibility that with shorter ambulation distances and always having a place to rest, the bones tolerate loads without acquiring microtrauma which lead to frank fractures. Despite these possibilities, based on the results of the present study, we can only conclude that the incidence of PFFs was reduced and not deduce to the mechanism and etiology of this reduction.

The Covid-19 pandemic caused unique challenges to the Israeli health systems and hospitals, as was the effect on health systems worldwide. However, the effect on the general population and especially on the elderly population was also substantial. The health system experienced the impact on the workforce, hospital ward allocation, and health system resources. New specialized Covid-19 hospital wards were opened, physicians and nursing staff were rearranged, and the increasing number of patients with more severe presentation caused increased burnout among hospital staff members [39, 40]. For the elderly population, these experienced increased vulnerability, as support systems were overworked and undermanned. This vulnerability, along with fear of contracting Covid-19 and imposed government lockdowns, led to increased loneliness, fear of going outside, and fear of reaching for medical assistance.

For this reason, we chose to focus on PFF as an archetypal fragility fracture and not on other fragility fractures such as distal radius fractures, proximal humerus fractures, and vertebral compression fractures. Patients sustaining these other fragility fractures may have been more likely to seek medical treatment at local clinics or even forego medical treatment altogether. In contrast, patients with PFFs would necessarily be treated at the tertiary medical center, and therefore the incidence found in this study reflects more accurately the actual number of cases occurring.

This study is not without limitations. First, we did not evaluate the effect of Covid-19 on the quality of treatment received by the patients. This can be evaluated using mortality rates as well as other parameters such as length of hospitalization, time to surgery, and others. However, while several countries reported severe healthcare crises, this was not a characteristic of the pandemic in Israel during 2020, although the first months of 2021 have shown an increased burden on the healthcare system. The inclusion criteria for our study were above 65 years old. There may be different results for the 65–75 group age as the population at this group of ages may be more active than 75 years old and above. A further limitation is that this study was conducted in one tertiary hospital; data was not available for other hospitals. A future national registry study may evaluate impact on a more extensive network of medical centers; thus, this may not reflect the situation at other medical centers or other regions of the country or world. A third limitation is a reliance on ICD-9 coding and the retrospective nature of the study. Lastly, although the study spans over ten years, as the pandemic continues to spread and cause morbidity globally into 2021, longer-term studies may be possible in the future.

In conclusion, we observed a total decrease in the number of PFF fractures occurring during 2020–21 compared to 2010–2019 and specifically a decrease of cases during the government-imposed lockdown periods. The decrease in cases was especially pronounced during the second and third lockdown periods. This study does not explain the mechanism of the trauma but suggests that trauma happens in unfamiliar territory. Thus, further studies regarding the specific mechanism should be conducted on the national level.

## Data Availability

The datasets generated and analysed during the current study are not publicly available as they are part of another ongoing research but are available from the corresponding author (Dr. Maria Oulianski, oulianskim@gmail.com) on reasonable request.

## Abbreviations

WHO: World Health Organization
MOH: Israeli Ministry of Health
ARDS: Acute respiratory distress syndrome
PFFs: Proximal femur fractures
STROBE: STrengthening the Reporting of OBservational studies in Epidemiology
ED: Emergency department
ICD-9: International Classification of Diseases, 9th edition
HDX: Humanitarian Data Exchange
SPSS: Statistical Product and Service Solutions
